# Efficacy of transcranial electrical stimulation in the treatment of chronic nonspecific low back pain: a systematic review and meta-analysis

**DOI:** 10.3389/fneur.2026.1797108

**Published:** 2026-08-19

**Authors:** Yuchen Zhou, Yifei Shou, Sitong Wang, Junqi Jia, Chuang Gao, Yufei Fang, Zhiqiang Liang

**Affiliations:** 1 Faculty of Sports Science, Ningbo University, Ningbo, China; 2 Department of Rehabilitation Medicine, Ningbo No. 2 Hospital, Wenzhou Medical University, Ningbo, China

**Keywords:** chronic low back pain, functional impairment, meta-analysis, pain intensity, psychological outcomes, systematic review, transcranial electrical stimulation

## Abstract

**Introduction:**

Chronic nonspecific low back pain (CNLBP) is a prevalent condition associated with substantial disability and a marked reduction in quality of life. First‑line treatments, such as pharmacotherapy and physical therapy, often have limited effectiveness. This systematic review and meta‑analysis evaluated the efficacy of transcranial electrical stimulation (tES) modalities, including transcranial direct current stimulation (tDCS), high‑definition transcranial direct current stimulation (HD‑tDCS), and transcranial alternating current stimulation (tACS), for the treatment of CNLBP.

**Methods:**

A comprehensive search was conducted across six electronic databases, and 19 randomized controlled trials met the inclusion criteria. Of these, 15 studies evaluated conventional tDCS, two evaluated high‑definition tDCS, one evaluated high‑definition transcranial infraslow pink‑noise stimulation (HD‑tIPNS), and one evaluated tACS.

**Results:**

The pooled results showed that single‑session tDCS did not significantly reduce pain intensity compared with sham. However, when combined with physical or behavioral interventions, tDCS was associated with significant pain reduction. Subgroup analyses showed no significant differences in outcomes according to stimulation target, including the primary motor cortex and dorsolateral prefrontal cortex.

**Discussion:**

Although tDCS showed promise as an adjunct for pain relief, its effects on functional disability and psychological outcomes were less conclusive. Further high‑quality studies are needed to optimize stimulation protocols and clarify the role of tDCS as an adjunct to other therapeutic approaches.

**Systematic review registration:**

https://www.crd.york.ac.uk/prospero/display_record.php?ID=CRD420261298298, CRD420261298298.

## 1 Introduction

Chronic nonspecific low back pain (CNLBP) is one of the leading contributors to disability-adjusted life years worldwide, imposing a substantial burden on healthcare systems and significantly impairing patients’ quality of life and functional status (1). Despite the widespread use of first-line interventions, such as pharmacotherapy and physical therapy, their effectiveness is often limited, and some patients experience poor outcomes or recurrence (2). One reason for this clinical challenge is that the pathophysiology of CNLBP frequently involves central sensitization and maladaptive neural plasticity, rather than being attributable solely to peripheral tissue abnormalities (3, 4).

Neuroimaging studies have shown that CNLBP is closely associated with functional and structural alterations in brain networks involved in sensation, emotion, and cognition, such as the default mode network and salience network (5). These central nervous system changes provide a theoretical basis for the use of non-invasive brain stimulation techniques to directly modulate cortical excitability and thereby alleviate pain (6). Among these approaches, transcranial electrical stimulation (tES) applies weak electrical currents to modulate neuronal membrane potentials and includes several modalities, such as transcranial direct current stimulation (tDCS), high-definition transcranial direct current stimulation (HD-tDCS), and transcranial alternating current stimulation (tACS) (7, 8).

In theory, stimulation of the primary motor cortex (M1) or dorsolateral prefrontal cortex (DLPFC) may exert therapeutic effects by modulating descending pain inhibitory pathways or influencing the emotional and cognitive dimensions of pain (9). However, the clinical efficacy of these approaches remains inconclusive. A Cochrane systematic review of chronic pain conditions suggested that, although tDCS may alleviate pain, the overall quality of the evidence is limited by study heterogeneity and methodological shortcomings, resulting in insufficient certainty regarding its clinical efficacy (10). For example, Alwardat et al. found that pooled analyses of available studies did not confirm significant improvements in pain or function in patients with CNLBP following tDCS compared with sham (11).

These inconsistencies suggest that tES is unlikely to be a universally effective intervention and that its efficacy may be influenced by several key factors. First, stimulation protocol parameters, particularly the number of sessions and current intensity, may substantially affect neural responses. A single session may induce only acute neuromodulatory effects, whereas repeated stimulation may involve more complex neural adaptation processes, or even tolerance (8), an issue that has not yet been systematically examined in CNLBP. Second, in clinical practice, tES is often delivered as part of a multimodal treatment strategy. However, whether and how it interacts synergistically with interventions such as exercise therapy, manual therapy, or cognitive behavioral therapy (CBT) remains unclear, and current findings are mixed. Importantly, improvements in pain intensity do not necessarily translate into recovery of daily function, and the relationship between these outcomes remains poorly understood.

Despite the growing body of literature on tES for CNLBP, several important questions remain unresolved. Previous meta-analyses have failed to distinguish between standalone and combined interventions, have focused mainly on tDCS rather than newer modalities such as HD-tDCS and tACS, and have not systematically evaluated functional disability and pain-related psychological outcomes. Therefore, the present study aimed to conduct a comprehensive and updated systematic review and meta-analysis to evaluate the efficacy of tES in patients with CNLBP. Specifically, we compared the effects of tES administered as a standalone intervention with those of tES combined with different physical therapeutic approaches; examined whether treatment effects differed according to stimulation target (M1 vs. DLPFC); and assessed the effects of tES on functional disability and pain-related psychological outcomes. By addressing these objectives, this review seeks to clarify the clinical utility of tES in the management of CNLBP, inform evidence-based practice, and guide the design of future trials.

## 2 Methods

This systematic review and meta-analysis were designed and reported in accordance with the Preferred Reporting Items for Systematic Reviews and Meta-Analyses (PRISMA) statement (12). This systematic review was prospectively registered on the International Prospective Register of Systematic Reviews (PROSPERO; registration number: CRD420261298298).

### 2.1 Search strategy

A comprehensive and systematic search was conducted across six Chinese and English electronic databases, namely PubMed, Web of Science, ScienceDirect, China National Knowledge Infrastructure, Wanfang Data, and the Chinese Quarterly Virtual Information Platform, from database inception to November 15, 2025. The search strategy combined free-text terms and controlled vocabulary, including Medical Subject Headings, where applicable, and used the terms ‘transcranial electrical stimulation,’ ‘transcranial direct current stimulation,’ ‘tDCS,’ ‘transcranial alternating current stimulation,’ ‘tACS,’ ‘transcranial random noise stimulation,’ ‘tRNS,’ ‘low back pain,’ ‘non-specific chronic low back pain,’ ‘chronic low back pain,’ ‘LBP,’ and ‘CLBP,’ as well as their corresponding Chinese terms. Complete search strings for all six databases, including all tES modality terms, are provided in Appendix 1.

### 2.2 Eligibility criteria

Eligibility criteria were established according to the PICOS framework. Studies were eligible if they included adults (≥18 years) with CNLBP, defined as pain lasting longer than 3 months; evaluated any form of tES, including but not limited to tDCS, HD-tDCS, and tACS, applied either as a standalone intervention or in combination with other therapies; used sham stimulation, no intervention, usual care, placebo control, or other methodologically comparable control conditions; and reported change in self-reported pain intensity at the end of treatment as the primary outcome, with low back pain-related functional impairment and pain-related psychological states assessed by standardized scales as secondary outcomes. Only randomized controlled trials (RCTs) published in English or Chinese, including both parallel-group and crossover designs, were included.

No prespecified minimum sample size or follow-up duration was imposed, as this review aimed to comprehensively capture the existing literature on the effects of tES on pain and related disability in CNLBP. Studies were excluded if they included participants with chronic pain of specific etiologies, such as neuropathic pain, fibromyalgia, or radicular pain, rather than nonspecific CNLBP; evaluated invasive brain stimulation or non-electrical non-invasive brain stimulation techniques, such as repetitive transcranial magnetic stimulation; or were published as conference abstracts, study protocols, reviews, letters, case reports, theses, or book chapters.

### 2.3 Study selection

All retrieved records were imported into EndNote 9.0 (USA) for record management and duplicate removal. Study selection was performed in two stages: first, two reviewers independently screened titles and abstracts to exclude obviously irrelevant studies; second, the full texts of potentially eligible studies were retrieved and assessed against the predefined inclusion and exclusion criteria. In addition, the reference lists of relevant systematic reviews and included studies were manually searched to identify any potentially eligible studies not captured through the electronic database search. Any disagreements were resolved through discussion between the two reviewers or, when necessary, consultation with a third senior researcher.

### 2.4 Data extraction

Data were independently extracted by two researchers using a predesigned form. Extracted information included study characteristics (authors, publication year, study design, sample size, and pain duration), intervention details (tES modality, stimulation target, electrode parameters, stimulation intensity, session duration, treatment frequency, total treatment duration, and co-interventions), control conditions, and outcome measures with corresponding assessment time points. For quantitative synthesis, means, standard deviations, and sample sizes at the primary post-intervention time point were extracted. When studies reported follow-up assessments, we prioritized the longest available follow-up time point for analysis. Most trials assessed outcomes immediately after the final session or within a short follow-up window (typically one month or less), and very few trials included extended follow-up periods (e.g., 3 months or longer). When necessary, these data were derived from change scores, standard errors, confidence intervals, or figures. For crossover trials, data were handled with consideration of the study design to avoid unit-of-analysis errors. Any discrepancies were resolved through discussion or consultation with a third researcher.

### 2.5 Risk of bias assessment

**Figure 1:**
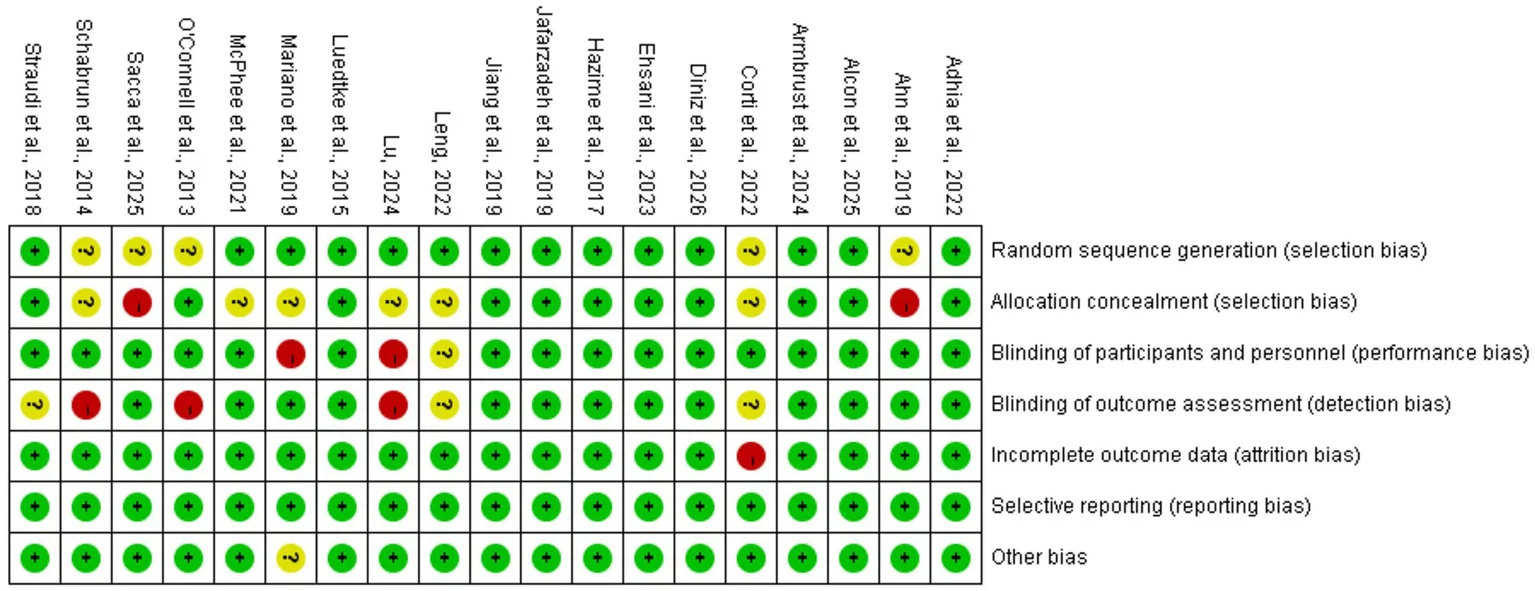
The risk of bias summary.

Risk of bias was independently assessed by two researchers using the original Cochrane Risk of Bias Tool (RoB 1), covering random sequence generation, allocation concealment, blinding of participants and personnel, blinding of outcome assessors, incomplete outcome data, selective reporting, and other potential sources of bias. Each domain was judged as low, high, or unclear risk based on the information reported in the original studies. Any disagreements were resolved through discussion or consultation with a third researcher. The final risk of bias assessments are presented in Figure 1.

### 2.6 Data synthesis and meta-analysis

All statistical analyses were performed using Review Manager version 5.4.1. Pain intensity, functional impairment, and pain-related psychological outcomes were analyzed. Weighted mean differences were used when the same assessment tool was applied across studies, whereas standardized mean differences were used when different instruments measured the same construct, with 95% confidence intervals (CIs) reported for all effect estimates. Heterogeneity was assessed using the I^2^ statistic; fixed-effects models were applied when I^2^ ≤ 50%, and random-effects models were used when I^2^ exceeding 50%. Subgroup analyses were performed according to tES modality and intervention type (standalone vs. combined intervention). Where meta-analysis was not appropriate because of limited study numbers, substantial clinical heterogeneity, or differences in outcome measures, findings were synthesized narratively. Statistical significance was set at *p* < 0.05.

Certainty of evidence for primary and secondary outcomes was assessed using the Grading of Recommendations Assessment, Development and Evaluation (GRADE) approach (13). Two reviewers independently rated certainty across five domains: risk of bias, inconsistency, indirectness, imprecision, and publication bias. Certainty was rated as high, moderate, low, or very low, and summarized in a Summary of Findings table (Appendix 5). We did not perform formal assessment of reporting biases (e.g., funnel plots, Egger’s test) due to the limited number of studies included in most meta-analyses and the high statistical heterogeneity observed.

## 3 Results

### 3.1 Study selection

**Figure 2:**
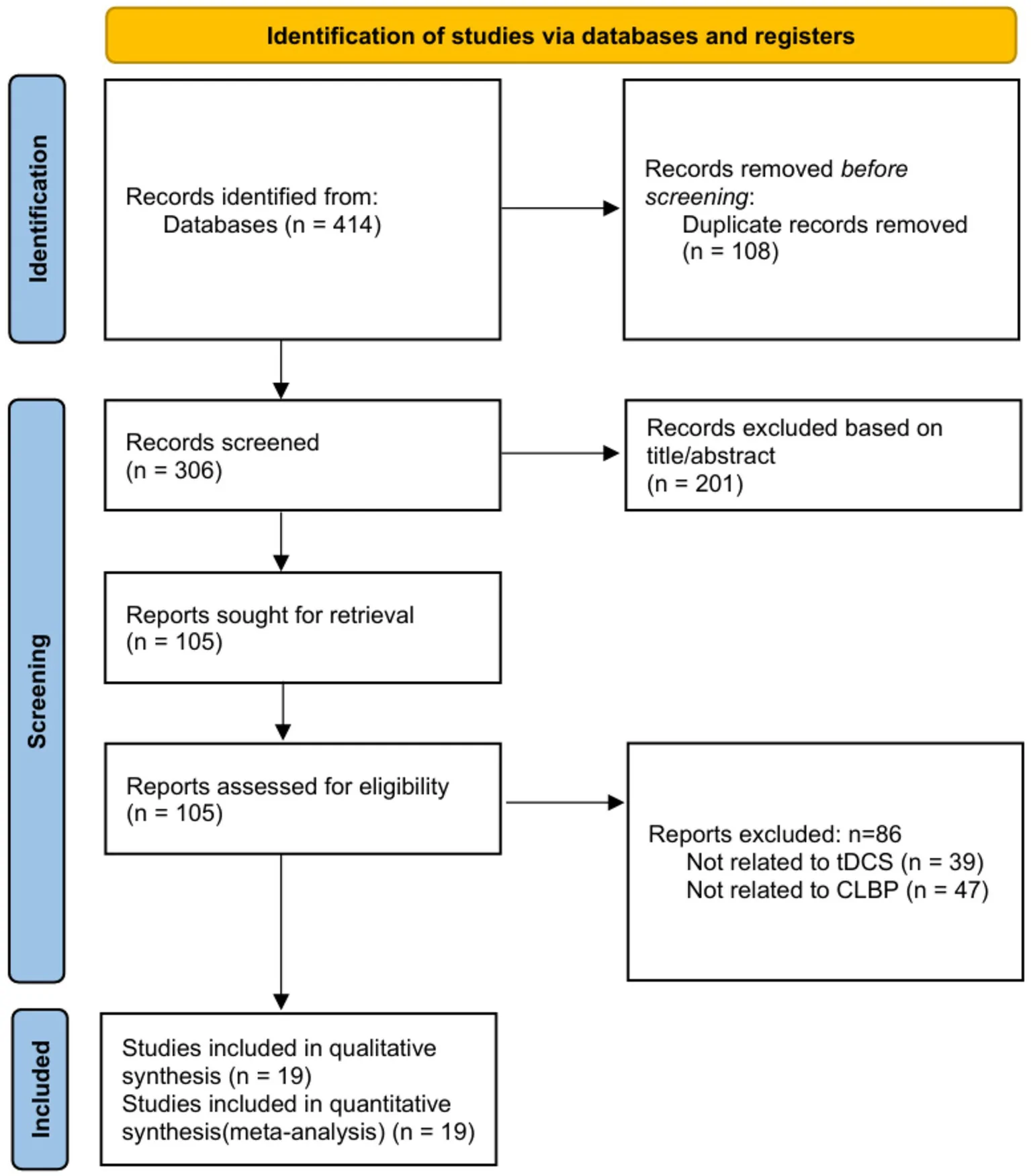
PRISMA flow diagram of study selection.

Study selection was conducted in accordance with the PRISMA guidelines, and the flow diagram is shown in Figure 2. The database search identified 414 records. After removal of 108 duplicates, 306 records were screened by title and abstract, and 201 were excluded. The full texts of 105 articles were assessed for eligibility, and 86 were excluded for not meeting the inclusion criteria. Ultimately, 19 trials were included in this systematic review.

### 3.2 Study characteristics

The characteristics of the included trials are summarized in Appendix 2.

#### 3.2.1 Participant characteristics

Across the included trials, 948 participants with CNLBP were enrolled. Sample sizes ranged from 8 to 122 participants per trial. Participants were aged 18 to 65 years. Among trials reporting mean age, the mean values ranged from 27.6 to 63.1 years. The largest trial was conducted by Luedtke et al. (*n* = 122) (14), whereas the smallest was conducted by O’Connell et al. (*n* = 8) (15).

#### 3.2.2 Study design characteristics

Of the 19 included trials, 15 used a parallel-group randomized controlled design and 4 used a crossover randomized controlled design (15–18). All crossover trials reported a washout period of at least 7 days.

#### 3.2.3 Stimulation intervention protocols

tDCS was the most frequently used intervention, appearing in 17 trials, including two HD-tDCS protocols (17, 19). Other electrical stimulation modalities relevant to this review were also investigated, including tACS (16) and HD-tIPNS (20). The number of stimulation sessions varied substantially, ranging from single-session (18, 21) protocols in two trials to as many as 12 sessions (22, 23) in two trials. Protocols involving 10 sessions (24–26) and 5 sessions (14, 27–29) were also relatively common.

Among the tDCS trials, the M1 was the most common stimulation target (14–16, 18–25, 27, 28, 30), followed by the DLPFC (25, 29, 31, 32). Additional targets included the dorsal anterior cingulate cortex (dACC) (26) and the medial prefrontal cortex (mPFC) (17). A current intensity of 2 mA was used in most trials (14–17, 19–30, 32, 33), whereas 1 mA (18) and 1.5 mA (31) were used less frequently. The stimulation duration was typically 20 min (14–17, 19–24, 26–33), with 30 min used in two trials (18, 25). Anodal stimulation was used in most trials (14–25, 27–31, 33), whereas two trials used cathodal stimulation (26, 32). The treatment dose ranged from a single session to 12 sessions, and treatment frequency varied across studies, including once-daily stimulation and two to three sessions per week.

#### 3.2.4 Primary outcomes

Nine instruments were used: the Visual Analogue Scale (VAS), Numeric Rating Scale (NRS), Brief Pain Inventory (BPI) (25), pain subscale of the RAND 36-Item Health Survey (14), Short-Form McGill Pain Questionnaire (SF-MPQ) (22), Defense and Veterans Pain Rating Scale (DVPRS) (20, 26), McGill Pain Questionnaire (MPQ) (17), and West Haven–Yale Multidimensional Pain Inventory (WHY-MPI-C). The NRS and VAS were each used in eight trials (NRS: 18–20, 22, 26–27, 29–30; VAS: 14–15, 17, 21, 24–25, 28, 31).

Disability outcomes were assessed in tACS (16) and tDCS trials using two instruments: the Roland–Morris Disability Questionnaire and the Oswestry Disability Index, including its modified version (MODI). The Roland–Morris Disability Questionnaire was used in 10 trials (15, 17, 22, 24–28, 31, 32), whereas the Oswestry Disability Index/MODI was used in 3 trials (14, 23, 29).

Pain-related psychological outcomes were assessed only in tDCS trials using four instruments: the Pain Catastrophizing Scale (PCS) (23, 29, 31), Tampa Scale of Kinesiophobia (TSK) (29, 32), Pain Anxiety Symptoms Scale (PASS) (26, 32), and Chronic Pain Acceptance Questionnaire-8 (CPAQ-8) (26).

Additional outcomes included behavioral, cognitive, brain-function, safety, and patient-reported measures; details are provided in Appendix 3.

### 3.3 Effects of transcranial electrical stimulation on pain in chronic non-specific low back pain

#### 3.3.1 Effect of transcranial direct current stimulation on pain intensity

**Figure 3:**
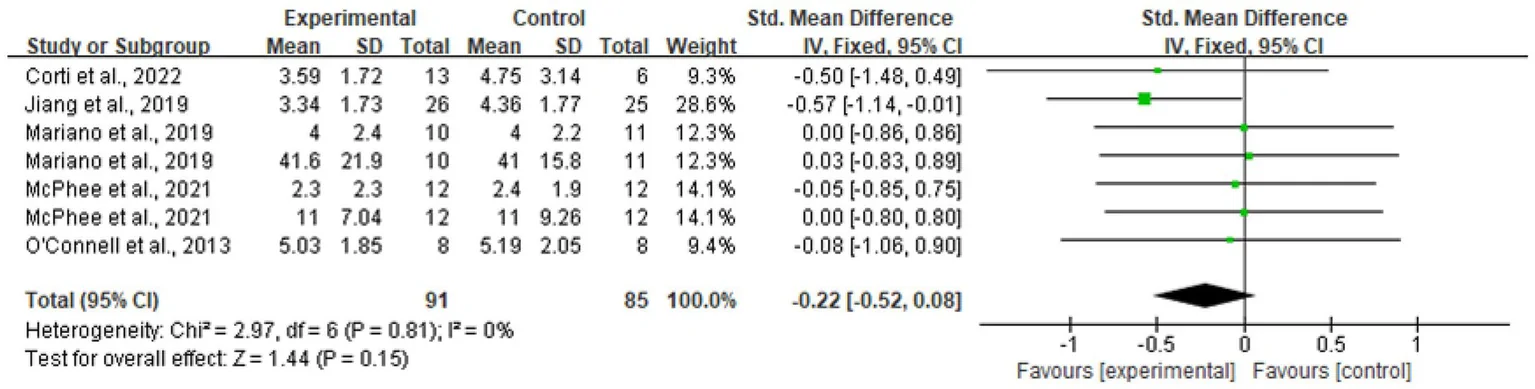
Forest plot of the effect of tDCS on pain intensity.

Five RCTs involving 176 participants (tDCS: 91; sham: 85) were eligible for pooling (Figure 3) (15, 17, 21, 26, 31). The seven trials from five studies were extracted into the fixed-effect meta-analysis. The pooled effect showed that no significant difference between tDCS and sham in improvement of pain intensity was found (SMD = −0.22, 95% CI [−0.52, 0.08], Z = 1.44, *p* = 0.15).

The eligible five studies, seven trials, targeted four cortical stimulation sites: 1 trial for DLPFC (31), 1 trial for mPFC (17), 1 trial for dACC (26), and 2 trials for M1 (15, 21). However, due to each site-specific stimulation sites contained trials was lower than three studies, subgroup meta-analysis was not performed.

The results by synthesized narratively showed that the single-season tDCS targeted on DLPFC, mPFC, dACC, and M1 did not produce the significant analgesic benefit over sham (Table 1).

**Table 1:** Summary of tDCS parameters for pain intensity.

Author	Number of sessions and frequency	Stimulated cortical target	Modality	Intensity (mA)	Stimulation duration (min)
Corti and Loftus, 2022 (31)	2 times/week, total of 4 weeks, total of 8 sessions	DLPFC	Anodal	1.5	20
Mariano et al., 2019 (26)	10 consecutive workdays, 1 session per day, total of 10 sessions	Left dACC	Cathodal	2	20
Mcphee et al., 2021 (17)	3 consecutive days, 1 session per day, total of 3 sessions	mPFC	Anodal	2	20
O’Connell et al., 2013 (15)	Average 9 sessions	M1	Anodal	2	20
Jiang et al., 2019 (21)	Single session	M1	Anodal	2	20

In O’Connell et al. (15), all participants received sham first and switched to daily active stimulation at a randomized time point (between Days 1 and 15) until study completion. The mean number of active sessions was 9 (range: 3–14). In this table, DLPFC is the dorsolateral prefrontal cortex; dACC is the dorsal anterior cingulate cortex; mPFC is the medial prefrontal cortex; M1 is the primary motor cortex.

#### 3.3.2 Effect of transcranial direct current stimulation combined with different therapies on pain in chronic non-specific low back pain

**Figure 4:**
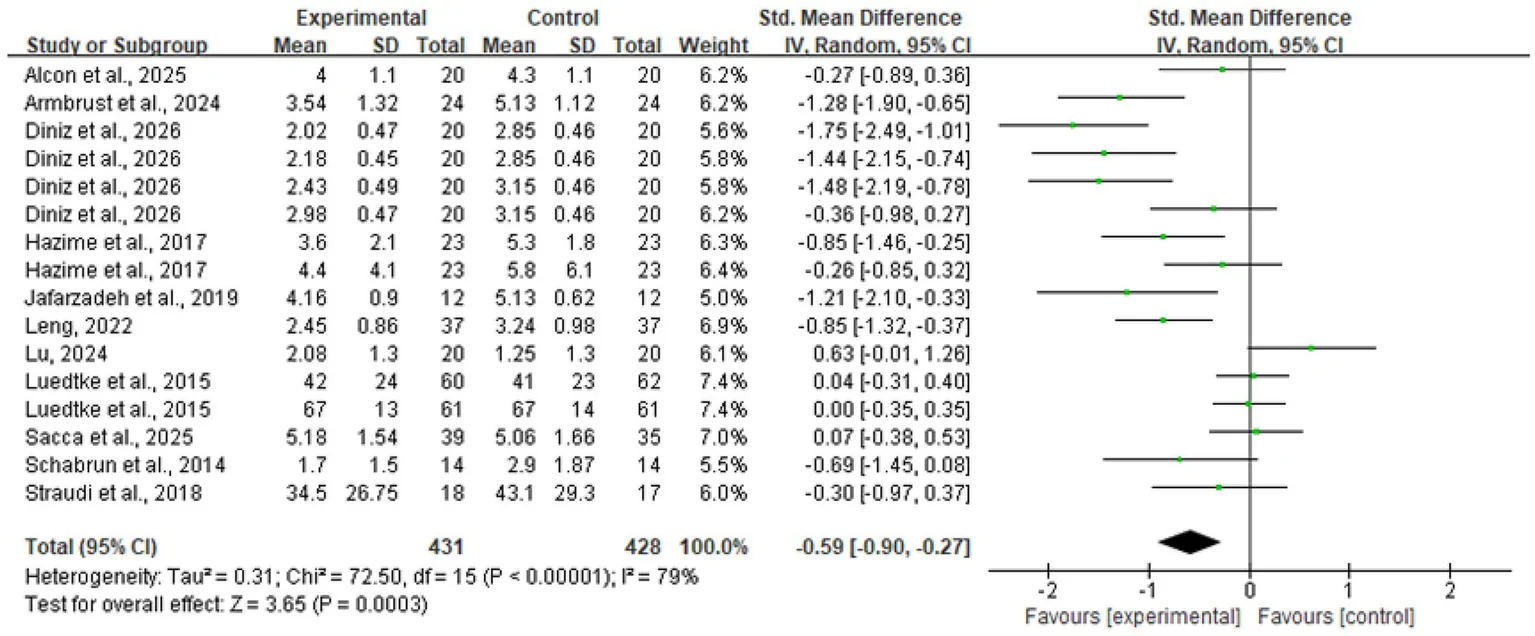
Forest plot of the effect of tDCS combined with different physical therapies on pain intensity.

Eleven RCTs involving 859 participants (tDCS: 431; sham/control: 428) were pooled (Figure 4) (14, 18, 19, 21, 23–25, 27–30). A random-effects model showed that tDCS combined with the therapies significantly reduced pain intensity compared with control group (SMD = −0.59, 95% CI [−0.90, −0.27], Z = 3.65, *p* = 0.0003).

**Figure 5:**
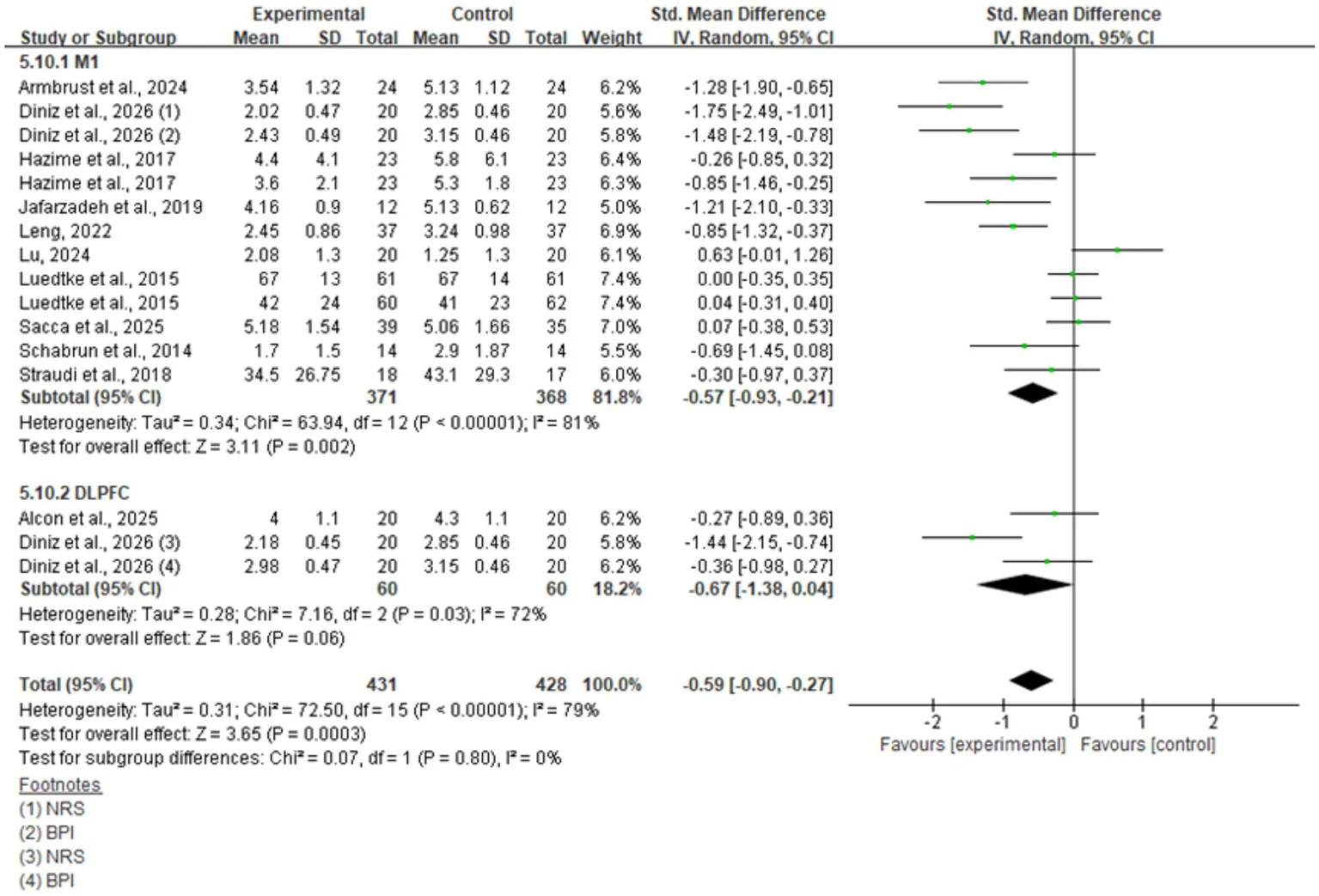
Subgroup analysis of tDCS combined therapies on pain intensity.

The pooled estimate for combined tDCS aggregated trials pairing stimulation with heterogeneous co-interventions (Figure 4). In the M1 subgroup (Figure 5), these included exercise therapy (23, 27, 28) osteopathic manipulative treatment (24), transcutaneous electrical nerve stimulation (TENS) (25), peripheral electrical stimulation (PES) (18, 22), postural training (30), acupuncture (19), and CBT (14). In the DLPFC subgroup (Figure 5), tDCS was paired with pain neuroscience education (29), TENS (25) and CBT (32). These co-interventions engage distinct therapeutic mechanisms: exercise, TENS, PES, and osteopathic manipulative treatment primarily target sensorimotor and peripheral pathways, whereas CBT, pain neuroscience education, and cognitive bias modification target cognitive-affective and educational dimensions. The pooled estimate therefore represents an average across functionally distinct combinations rather than the specific efficacy of any single pairing.

In the M1 subgroup (14, 18, 19, 22–25, 27, 28, 30), tDCS combined with 10 therapies, including exercise therapy, acupuncture, PES, TENS, osteopathic manipulative treatment, and CBT, to relieve pain intensity (Figure 5). Subgroup analysis showed that tDCS targeted M1 combined therapies significantly relieve pain intensity (SMD = −0.57, 95% CI [−0.93, −0.21], *Z* = 3.11, *p* = 0.002), with the higher within-subgroup heterogeneity (*I*^2^ = 81%). Difference with M1 subgroup analysis, in the DLPFC subgroup (25, 29), tDCS over DLPFC combined with the therapies did not significantly relieve pain intensity in CNLBP patient (SMD = −0.67, 95% CI [−1.38, 0.04], *Z* = 1.86, *p* = 0.06), with higher heterogeneity (*I*^2^ = 79%). A random-effects model (*I*^2^ = 79%) was further used to analyze subgroup difference, there is no significant difference between tDCS over M1 and tDCS over DLPFC in relieving pain intensity in CNLBP patient (χ^2^ = 0.07, *p* = 0.80) (Table 2).

**Table 2:** Summary of tDCS parameters for pain intensity.

Author	Number of sessions and frequency	Stimulated cortical target	Modality	Intensity (mA)	Stimulation duration (min)
Armbrust et al., 2024 (24)	10 consecutive workdays, 1 session per day, total of 10 sessions	M1	Anodal	2	20
Hazime et al., 2017 (22)	3 times/week, total of 4 weeks, total of 12 sessions	M1	Anodal	2	20
Jafarzadeh et al., 2019 (30)	3 times/week, total of 2 weeks, total of 6 sessions	M1	Anodal	2	20
Luedtke et al., 2015 (14)	5 consecutive days, 1 session per day, total of 5 sessions	M1	Anodal	2	20
Sacca et al., 2025 (19)	Total of 6 sessions	M1	Anodal	2	20
Schabrun et al., 2014 (18)	Total of 4 sessions, each intervention type once	M1	Anodal	1	30
Straudi et al., 2018 (28)	1 session per day for 5 consecutive days	M1	Anodal	2	20
Bo L., 2022 (27)	1 session per day for 5 consecutive days, total of 5 sessions	M1	Anodal	2	20
Lu et al., 2024 (23)	3 times/week, total of 4 weeks, total of 12 sessions	M1	Anodal	2	20
Diniz et al., 2026 (25)	10 consecutive workdays, 1 session per day, total of 10 sessions	M1	Anodal	2	30
		DLPFC			
Alcon et al., 2024 (29)	Completed all sessions within 2 weeks, total of 5 sessions	DLPFC	Anodal	2	20

DLPFC is the dorsolateral prefrontal cortex; M1 is the primary motor cortex.

The pooled SMD of −0.59 corresponds to a moderate standardized effect size. However, its clinical meaningfulness is difficult to determine because the included trials used heterogeneous pain scales and did not consistently report responder rates or the proportion of participants achieving minimal clinically important difference (MCID) thresholds. Moreover, the absolute change in pain scores corresponding to this standardized effect varied across studies depending on baseline severity and scale-specific standard deviations. Consequently, while the effect is statistically robust, whether it represents a perceptible and clinically meaningful improvement for patients with CNLBP remains uncertain.

#### 3.3.3 Effects of transcranial direct current stimulation on pain threshold

Three studies assessed pressure pain threshold (PPT) (17, 18, 29). Alcon et al. (29) and McPhee et al. (17) found that neither tDCS nor tDCS combined with pain neuroscience education (PNE) altered PPT. Schabrun et al. (18) reported that only tDCS combined with PES increased local PPT, while tDCS, PES, or sham were ineffective. One additional study by McPhee et al. (17) evaluated conditioned pain modulation, temporal summation, and cuff algometry, reporting no significant between-group differences in descending pain inhibition, central sensitization markers, or deep-tissue pain sensitivity following HD-tDCS.

#### 3.3.4 Effects of rest of transcranial electrical stimulation on pain intensity

The rest of two studies analyzed the effect of HD-tIPNS and tACS on pain intensity, via stimulating the DLPFC and pain-related brain network consisting of perigenual anterior cingulate cortex, dACC, and bilateral S1. Adhia et al. (20) found a single session of HD-tIPNS significantly modulated pain-related brain network connectivity, but did not produce clinical analgesia.

Ahn et al. (16) found that single-session tACS at 10 Hz applied over the bilateral somatosensory cortex (F3 and F4) significantly relieved pain intensity in CNLBP, via increased alpha oscillatory power in the sensorimotor cortex.

However, as each of these modalities was represented by only a single exploratory trial, the available evidence remains preliminary and is insufficient to draw firm conclusions regarding their clinical efficacy in CNLBP.

### 3.4 Effects of transcranial electrical stimulation on functional disability in chronic non-specific low back pain

#### 3.4.1 Effect of transcranial direct current stimulation on functional disability

**Figure 6:**
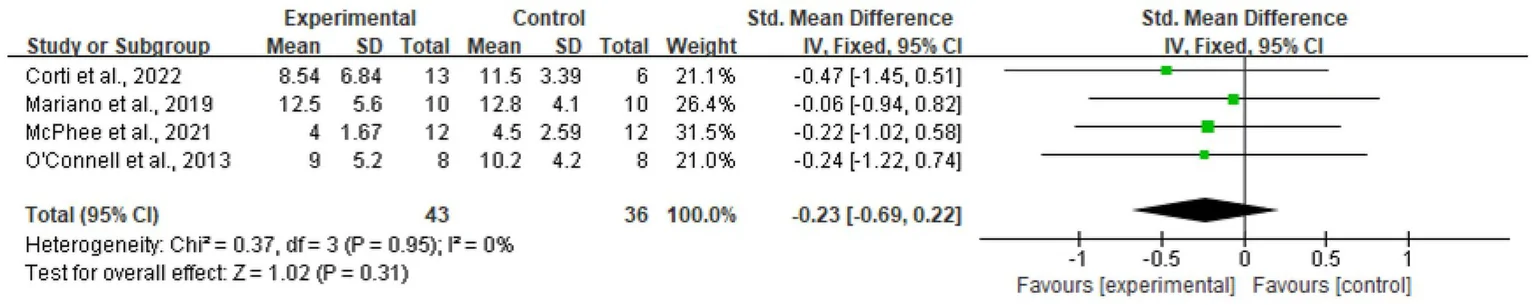
Forest plot of the effect of tDCS on functional disability.

Four RCTs involving 79 participants (tDCS: 43; sham: 36) were pooled (Figure 6). A fixed-effect model showed, tDCS targeted DLPFC (31), left dACC (26), mPFC (17), and M1 (15) did not significantly improve the functional disability than sham (SMD = −0.23, 95% CI [−0.69, 0.22], Z = 1.02, *p* = 0.31) (Table 3).

**Table 3:** Summary of tDCS parameters for functional disability.

Author	Number of sessions and frequency	Stimulated cortical target	Modality	Intensity (mA)	Stimulation duration (min)
Corti and Loftus, 2022 (31)	2 times/week, total of 8 sessions	DLPFC	Anodal	1.5	20
Mariano et al., 2019 (26)	10 consecutive workdays, 1 session per day, total of 10 sessions	dACC	Cathodal	2	20
Mcphee et al., 2021 (17)	3 consecutive days, 1 session per day, total of 3 sessions	mPFC	Anodal	2	20
O’Connell et al., 2013 (15)	Average 9 sessions	M1	Anodal	2	20

dACC is the dorsal anterior cingulate cortex; mPFC is the medial prefrontal cortex.

#### 3.4.2 Effect of transcranial direct current stimulation combined with different therapies on functional disability in chronic non-specific low back pain

**Figure 7:**
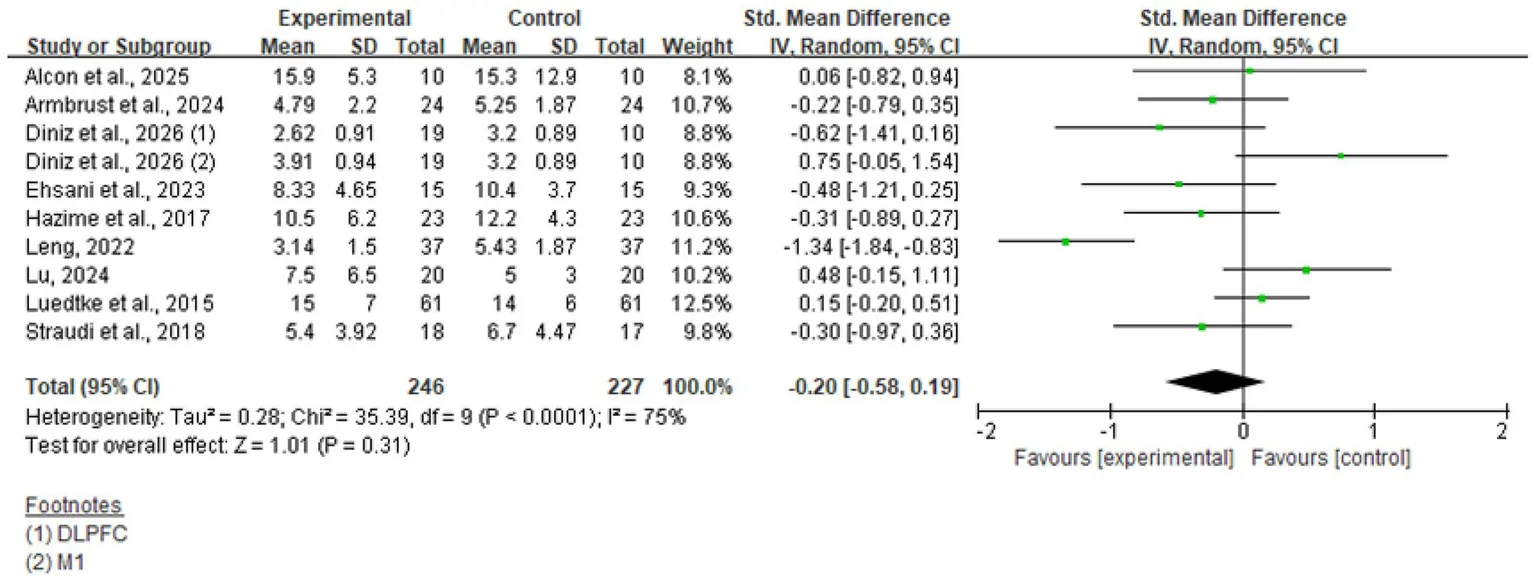
Forest plot of the effect of combined tDCS on functional disability.

Ten RCTs involving 473 participants (tDCS: 246; control: 227) were pooled (Figure 7). The higher heterogeneity was found (*I*^2^ = 75%), then a random-effects model was used. The pooled effect was not statistically significant (SMD = −0.20, 95% CI [−0.58, 0.19], *Z* = 1.01, *p* = 0.31).

**Figure 8:**
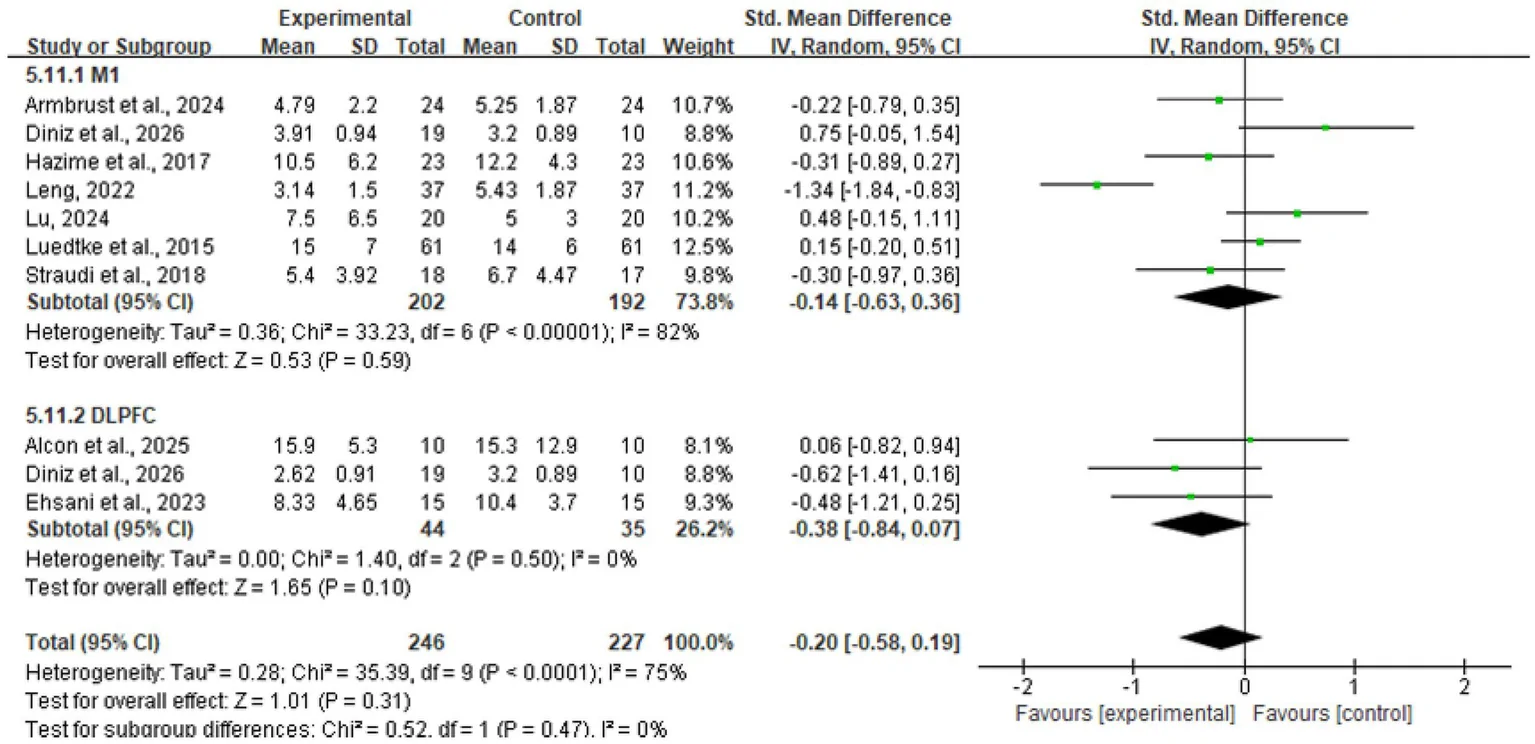
Subgroup analysis of combined tDCS on functional disability by stimulation target.

The functional disability analysis (Figure 7) aggregated trials with heterogeneous cointerventions. In the M1 subgroup (Figure 8), these comprised exercise therapy (23, 27, 28), osteopathic manipulative treatment (24), TENS (25), PES (22), and CBT (14). In the DLPFC subgroup (Figure 8), tDCS was combined with pain neuroscience education (29), CBT (32), and TENS (25). These co-interventions target distinct mechanisms, from peripheral neuromodulation and motor rehabilitation to cognitive restructuring and pain education. The pooled estimate for functional disability therefore represents an average across mechanistically distinct pairings and may not reflect the efficacy of any specific combination.

Subgroup analyses stratified by comparing the cortical differences showed no statistically significant between-subgroup difference (χ2 = 0.52, *p* = 0.47) (Figure 8). Results in the M1 subgroup consisting of 7 trials, including 402 participants, did not show the significant improvement of tDCS on functional disability (SMD = −0.14, 95% CI [−0.63, 0.36], Z = 0.53, *p* = 0.59), with high heterogeneity (I^2^ = 82%). In the DLPFC subgroup consisting of 3 trials, 88 participants, did also not show the significant improvement of tDCS on functional disability (SMD = −0.38, 95% CI [−0.84, 0.07], Z = 1.65, *p* = 0.10) (Table 4).

**Table 4:** Summary of tDCS parameters for functional disability.

Author	Number of sessions and frequency	Stimulated cortical target	Modality	Intensity (mA)	Stimulation duration (min)
Armbrust et al., 2024 (24)	10 consecutive workdays, 1 session per day, total of 10 sessions	M1	Anodal	2	20
Hazime et al., 2017 (22)	3 times/week, total of 4 weeks, total of 12 sessions	M1	Anodal	2	20
Luedtke et al., 2015 (14)	5 consecutive days, 1 session per day, total of 5 sessions	M1	Anodal	2	20
Straudi et al., 2018 (28)	1 session per day for 5 consecutive days	M1	Anodal	2	20
Bo L., 2022 (27)	1 session per day for 5 consecutive days, total of 5 sessions	M1	Anodal	2	20
Lu et al., 2024 (23)	3 times/week, total of 4 weeks, total of 12 sessions	M1	Anodal	2	20
Diniz et al., 2026 (25)	10 consecutive workdays (Monday to Friday), 1 session per day, total of 10 sessions	M1	Anodal	2	30
		DLPFC			
Alcon et al., 2024 (29)	Completed all sessions within 2 weeks, total of 5 sessions	DLPFC	Anodal	2	20
Ehsani et al., 2023 (32)	2 times/week, total of 4 weeks, total of 8 sessions	DLPFC	Cathodal	2	20

#### 3.4.3 Effects of rest of transcranial electrical stimulation on functional disability

Compared with tDCS, only one study assessed the tACS effect on functional disability (16). Results did not find the significant improvement in functional disability after a single session of 10 Hz tACS compared with sham.

Given that this finding derives from a single small-scale pilot study, it should be regarded as preliminary and cannot be generalized to support the efficacy of tACS for functional disability in CNLBP.

### 3.5 Effects of transcranial direct current stimulation on psychological outcomes

Five eligible studies examined psychological outcomes in patients with CNLBP using questionnaire-based measures assessing pain catastrophizing, kinesiophobia, pain anxiety, and pain acceptance. Owing to the small number of included trials and the heterogeneity of outcome measures, a quantitative meta-analysis was not feasible; therefore, psychological outcomes were synthesized narratively. The key characteristics and findings of these studies are summarized in Table 5.

**Table 5:** Narrative synthesis of the effects of tDCS on pain-related psychological outcomes.

Author	Modality	Psychological assessment tools	Key findings
Corti et al., 2022 (31)	tDCS	PCS	Did not effectively improve pain scores
Alcon et al., 2024 (29)	tDCS+PNE	PCS、TSK	Combined benefits observed only for pain catastrophizing (PCS), no combined benefit for TSK
Ehsani et al., 2023 (32)	tDCS+CBT	PASS、TSK	Strong combined benefits with long-lasting effects
Lu et al., 2024 (23)	tDCS+sprot	PCS	No combined benefits observed
Mariano et al., 2019 (26)	tDCS	PASS、CPAQ-8	Did not effectively improve pain-related anxiety or pain acceptance

In this table, PCS is the Pain Catastrophizing Scale; TSK is the Tampa Scale for Kinesiophobia; PASS is the Pain Anxiety Symptoms Scale; CPAQ-8 is the Chronic Pain Acceptance Questionnaire-8; CBT is cognitive behavioral therapy.

Three studies assessed pain catastrophizing (23, 29, 31). Only one study showed that tDCS combined with PNE was associated with greater improvements in pain catastrophizing, whereas the remaining evidence did not demonstrate improvement (29). Taken together, the evidence is inconsistent, with one positive trial and two null trials, precluding a definitive conclusion regarding the effect of tDCS on pain catastrophizing. Two studies assessed kinesiophobia (29, 32), and two studies assessed pain anxiety (26, 32). Only one study reported that tDCS combined with CBT produced significant and sustained improvements in both kinesiophobia and pain anxiety, whereas the other studies found no improvement. Overall, the findings for fear-related constructs are conflicting, with one study showing benefit and others showing no effect, suggesting that any benefit may be highly dependent on the specific co-intervention and stimulation protocol. One study assessed pain acceptance and reported no statistically significant improvement with standalone tDCS (26). Given the single-study evidence, no conclusion can be drawn regarding pain acceptance.

#### 3.5.1 Other psychological scales

Beyond pain-specific psychological outcomes, studies used heterogeneous instruments to assess fear-avoidance, anxiety/depression symptoms, and affect; owing to sparse reporting and limited overlap in measures, findings are summarized narratively.

Three studies assessing depressive symptoms with the Patient Health Questionnaire-9 all reported positive effects of tDCS (26–28). Four studies using other psychological scales did not demonstrate superiority of tDCS over control groups in improving anxiety, depression, or emotional states (14, 17, 23, 26). Thus, the evidence for mood-related outcomes is mixed: positive findings were observed only with the Patient Health Questionnaire-9, whereas other instruments showed no group differences, indicating a lack of consistent evidence across measures.

### 3.6 Effects of transcranial electrical stimulation on other outcomes in chronic non-specific low back pain

Outcomes beyond pain and disability included sensorimotor, behavioral, cognitive, and neurophysiological/neurofunctional measures. Given the small number of studies contributing data to most outcome domains and the heterogeneity of assessment methods, these findings were synthesized narratively.

#### 3.6.1 Effects of transcranial electrical stimulation on sensorimotor, behavioral, and somatosensory outcomes

Findings for sensorimotor, behavioral, and somatosensory outcomes were mixed. In Jafarzadeh et al. (30), significant improvements were reported in both the Biodex stability indices and Berg Balance Scale scores. Schabrun et al. (18) found that only tDCS combined with PES significantly increased pain-free lumbar flexion. The same study also reported that both tDCS combined PES and PES significantly reduced two-point discrimination thresholds, with no significant difference between these two interventions. These findings suggest that tDCS may augment sensorimotor and behavioral outcomes when paired with active rehabilitation or peripheral stimulation, although the evidence is limited to few studies and lacks replication.

By contrast, electromyographic based neuromuscular activation outcomes were largely null. In Diniz et al. (25), transversus abdominis activation did not differ across time points. Similarly, Jiang et al. (21) found no significant improvement in muscle activation patterns with tDCS. Taken together, the evidence converges on a null effect for neuromuscular activation outcomes, with no study demonstrating a significant benefit of tDCS over control.

#### 3.6.2 Effects of transcranial electrical stimulation on cognitive outcomes

Evidence for cognitive outcomes was limited and inconsistent. Alcon et al. (29) reported that combined tDCS improved interference control and response inhibition, as measured by the Stroop Test and the CTMT-2 inhibition index, with large effect sizes; however, no between-group difference was observed for set-shifting. In contrast, O’Connell et al. (15) found no detectable between-group differences in cognitive task performance across multiple measures. Given these conflicting findings and the small number of studies, no reliable conclusion can be drawn regarding the effects of tDCS on cognitive function in CNLBP.

#### 3.6.3 Effects of transcranial electrical stimulation on neurophysiological and neurofunctional outcomes

Evidence for neurophysiological and neurofunctional outcomes showed modality-specific effects. Sacca et al. (19) reported condition-dependent changes in cerebral blood flow following both real tDCS and sham tDCS, as well as both real and sham acupuncture. In Adhia et al. (20), HD-tIPNS significantly modulated functional connectivity patterns compared with sham stimulation, reducing instantaneous connectivity and causal information flow between specific brain regions. One tACS study (16) reported that bifrontal tACS increased sensorimotor alpha power compared with sham, and this increase was associated with improvements in pain severity. Notably, the neurophysiological findings are discordant across modalities: tDCS produced non-specific cerebral blood flow changes, whereas HD-tIPNS and tACS showed more specific neural effects, but none translated consistently into clinical improvement. As each of these modalities was represented by only a single exploratory trial, the available evidence remains preliminary and is insufficient to draw firm conclusions regarding their clinical efficacy in CNLBP.

It should be emphasized that the above findings for tACS and HD-tIPNS derive from one study each, both with small sample sizes. Therefore, these observations are inferential and hypothesis-generating rather than constituting conclusive evidence of clinical efficacy. Any interpretations regarding the therapeutic potential of these non-tDCS tES modalities remain tentative until replicated in larger, well-controlled trials.

#### 3.6.4 Safety and tolerability

All 19 included trials reported monitoring of adverse events or tolerability outcomes. The most commonly reported adverse events associated with active tDCS were mild and transient cutaneous sensations, including tingling, itching, and mild discomfort under the electrodes, which typically resolved spontaneously during or shortly after stimulation. No serious adverse events were reported across the included trials. Dropout rates were generally low and comparable between active and sham or control groups, with most attrition attributed to scheduling conflicts or personal reasons unrelated to intervention tolerability. A minority of participants reported mild headache or fatigue, with no significant differences between active and sham conditions.

### 3.7 Certainty of evidence

We assessed the certainty of evidence for all primary and secondary outcomes using the GRADE approach. The certainty ratings and supporting rationale are summarized in Appendix 5. For the primary outcome of pain intensity, the certainty of evidence was rated as low for standalone tDCS versus sham (downgraded for risk of bias and imprecision) and very low for combined tDCS versus control/sham (downgraded for risk of bias, very serious inconsistency, and suspected publication bias). For functional disability, the certainty of evidence was very low for both standalone and combined tDCS (downgraded for risk of bias, imprecision, and inconsistency). The certainty of evidence for pain-related psychological outcomes, pressure pain threshold, and emerging modalities (tACS and HD-tIPNS) was rated as very low, reflecting very small study numbers, high heterogeneity, indirectness of outcome measures, and the exploratory nature of the available trials. These ratings indicate that the certainty of evidence for tDCS in CNLBP varies by outcome and intervention type: moderate certainty for standalone tDCS versus sham, but low to very low certainty for combined tDCS protocols and for all functional disability comparisons. This heterogeneity in evidence quality underscores the need for further high-quality, adequately powered trials to clarify the clinical utility of specific tDCS configurations.

## 4 Discussion

This systematic review and meta-analysis comprehensively evaluated the efficacy of tES for CNLBP. The main findings were as follows: standalone tDCS did not produce a significant analgesic effect compared with sham stimulation, whereas tDCS combined with co-interventions, such as CBT, physical therapy, or PNE, significantly reduced pain intensity. Whether administered alone or in combination, tDCS did not result in statistically significant improvements in disability. For psychological outcomes, combined interventions may confer some benefit for pain catastrophizing, but the findings for kinesiophobia and pain anxiety were inconsistent. Preliminary studies of other tES modalities, such as tACS and HD-tIPNS, suggested potential neurophysiological effects; however, these effects did not translate into clinical analgesia. Importantly, the differential findings across intervention groups may be attributable to differences in stimulation target, treatment dose, co-intervention type, and the extent to which the paired intervention actively engages the neural circuits modulated by tES. It should be emphasized that 17 of the 19 included trials evaluated conventional tDCS, with only single exploratory studies available for tACS and HD-tIPNS. Therefore, the pooled conclusions primarily reflect tDCS evidence, whereas findings for other tES modalities remain preliminary. Notably, tDCS and related tES modalities demonstrated a favorable safety and tolerability profile across the included trials, with only mild and transient adverse events reported. This supports the feasibility of tES as an adjunctive intervention in clinical settings, although larger trials with systematic adverse event reporting are needed to confirm long-term safety.

Although the pooled analysis demonstrated that combined tDCS significantly reduced pain intensity (SMD = −0.59), the clinical relevance of this effect requires careful interpretation. A standardized mean difference of approximately 0.6 is conventionally regarded as moderate; however, its clinical significance in CNLBP depends on whether the corresponding absolute change in pain scores meets established minimal clinically important difference (MCID) thresholds. The included trials employed diverse pain instruments (VAS 0–10, NRS 0–10, BPI 0–10), and the absolute reductions corresponding to this SMD ranged widely across studies. Furthermore, none of the included trials systematically reported responder analyses or the proportion of participants achieving predefined MCID thresholds, precluding a definitive judgment on whether the average reduction is perceptible and meaningful to patients. Future trials should incorporate anchor-based MCID definitions and patient-reported global impression of change to clarify the clinical value of these effects.

In addition, the current evidence base provides limited information on the durability of tDCS-induced analgesia. Most included trials assessed outcomes immediately after the final session or within a one-month follow-up window, and very few studies incorporated extended follow-up assessments (e.g., 3 months or longer). The chronic nature of CNLBP necessitates evidence on sustained benefit; without longer-term data, it remains uncertain whether the observed pain reductions represent transient neuromodulatory after-effects or durable clinical improvement. This absence of long-term evidence is a critical gap that future adequately powered RCTs with extended follow-up periods must address.

The absence of a significant analgesic effect with standalone tDCS is consistent with previous meta-analyses (34). One possible explanation is that the pathophysiology of CNLBP is more complex than that of acute nociceptive pain. Acute pain is typically associated with localized nociceptive input, whereas chronic pain, particularly CNLBP, involves central sensitization and widespread reorganization of brain networks (3, 4). tDCS modulates cortical excitability through weak electrical currents, and its effects are generally restricted to the stimulation site and its connected neural circuits (8). In contrast, chronic pain-related abnormalities often extend across distributed networks, including the default mode network, salience network, and sensorimotor system (5). It is therefore plausible that modulation of a single cortical target is insufficient to reverse entrenched network-level maladaptive plasticity. In addition, the standalone tDCS studies included in this review varied substantially in stimulation target and treatment dose. Although most used a conventional protocol of 2 mA for 20 min, the number of sessions ranged from one to ten, and targets included M1, DLPFC, dACC, and mPFC. Such variability may have diluted any target-specific effect, and the relatively limited number of sessions in several studies may have been insufficient to induce clinically meaningful carryover effects in an established chronic pain state (18). Thus, the lack of efficacy of standalone tDCS may reflect both a mismatch between focal neuromodulation and distributed pain pathology, and an insufficient cumulative stimulation dose. This explanation is consistent with current pain neuroscience theory, though the included studies did not acquire neuroimaging or connectivity data that would directly test this network-level hypothesis.

By contrast, tDCS significantly reduced pain intensity when combined with CBT, exercise therapy, physical therapy, or PNE. This pattern suggests that tDCS may function more effectively as an adjunctive neuromodulatory technique than as a standalone analgesic intervention. A possible mechanistic explanation is that the effects of tDCS are state-dependent and activity-dependent, such that stimulation may be more likely to produce clinically meaningful effects when delivered concurrently with interventions that actively recruit task-relevant neural circuits. In this context, tDCS may not directly eliminate pain, but may instead prime the nervous system to respond more effectively to behavioral or physical interventions by facilitating neuroplasticity within engaged networks.

This framework may also help explain differences across combined intervention groups. When tDCS is applied over the M1 in conjunction with exercise or physical therapy, the paired intervention directly engages sensorimotor circuits that are relevant to movement control and pain-related motor adaptations (6). It is conceivable that, under these conditions, tDCS could facilitate motor learning, promote more adaptive movement patterns, and reduce pain-related compensatory behaviors, thereby indirectly contributing to pain relief (18, 30). In contrast, when stimulation is applied over the DLPFC, one might expect that benefit may depend more strongly on whether the co-intervention effectively engages cognitive-affective processes such as attention regulation, cognitive control, and pain reappraisal (9, 35). If so, DLPFC stimulation would likely be more relevant to catastrophizing, pain-related distress, or maladaptive pain beliefs than to pain intensity per se. Consistent with this tentative interpretation, M1-targeted combined interventions appeared more consistently beneficial for pain, whereas the effect of DLPFC stimulation did not reach statistical significance in the subgroup analysis (*p* = 0.06). However, because only two studies examined DLPFC-based combined interventions for pain intensity, this account remains speculative and requires direct testing in future research (36). Importantly, there was no statistically significant difference between M1 and DLPFC subgroups, indicating that the apparent discrepancy may also reflect limited statistical power rather than a true target-specific difference. Collectively, these target-specific comparisons should be regarded as exploratory and hypothesis-generating rather than conclusive, and any inference regarding the superiority of one cortical target over another for CNLBP would be premature given the very limited number of DLPFC trials.

The pooled estimates in this review aggregate trials with considerable clinical and methodological diversity. Stimulation protocols differed in parameters known to influence neuromodulatory efficacy, including session number, current intensity, duration, polarity, and cortical target. Outcome instruments for pain also varied in their sensitivity and scaling properties. Consequently, the pooled effect sizes represent averages across functionally distinct interventions rather than precise estimates of any single protocol, and should be interpreted with caution.

The high heterogeneity observed in the combined-treatment analysis (I^2^ = 79%) further supports the view that the effectiveness of tDCS may depend on how it is paired with other interventions. The included studies differed substantially in the type, frequency, duration, and intensity of the co-interventions, as well as in stimulation target, session number, and participant characteristics. Such variability is highly relevant mechanistically, because the magnitude and direction of neuromodulatory effects may depend on the degree of temporal and functional coupling between brain stimulation and the behavioral process being trained. For example, Schabrun et al. (18) found that local pain thresholds improved only when tDCS was combined with PES, whereas neither standalone tDCS nor standalone peripheral stimulation was effective. This finding suggests that certain combinations may be capable of engaging complementary peripheral and central mechanisms, thereby producing effects that are not observed when either component is delivered in isolation.

Despite the reduction in pain with combined tDCS, disability did not improve significantly with either standalone or combined treatment. This dissociation is clinically important and may reflect the multidimensional nature of functional disability in CNLBP. Functional limitation is not determined solely by pain intensity, but is also influenced by muscle strength, joint mobility, motor control, physical deconditioning, fear-avoidance beliefs, and psychosocial factors (37, 38). As tDCS primarily targets central nervous system processes, it may be insufficient to directly restore peripheral musculoskeletal function or reverse long-standing movement dysfunction (39). Thus, although pain may improve through modulation of nociceptive or cognitive-emotional processes, these changes may not be sufficient to produce measurable gains in disability over the relatively short intervention and follow-up periods of most included studies. This may explain why analgesic effects were observed in some combined-treatment groups without parallel improvements in functional disability (40).

The narrative synthesis of psychological outcomes revealed a similarly differential pattern. tDCS combined with PNE or CBT may improve pain catastrophizing (29, 32), whereas its effects on kinesiophobia and pain anxiety were inconsistent. One possible explanation is that these psychological constructs do not rely on identical neural substrates or respond equally to the same stimulation target. Pain catastrophizing is more closely linked to maladaptive cognitive appraisal and prefrontal-limbic dysregulation, whereas kinesiophobia may be more strongly maintained by threat-related movement expectations, avoidance learning, and motor behavior (4). By extension, DLPFC stimulation might preferentially influence catastrophizing through its role in cognitive control and reappraisal, whereas kinesiophobia may require repeated behavioral exposure, movement retraining, or longer treatment duration to show comparable change. These interpretations draw on broader pain neuroscience theory and should be considered hypothesis-generating, as the included studies did not directly measure neural substrates or compare stimulation targets within the same psychological domain. Differences in stimulation polarity, session number, and the intensity of the paired psychological intervention may also have contributed to the inconsistent findings. For example, Ehsani et al. (32) used eight sessions of cathodal tDCS combined with CBT and observed sustained improvements in fear and anxiety, whereas Alcon et al. (29) used five sessions of anodal tDCS combined with PNE and observed benefit only for catastrophizing. Although these studies are not directly comparable, they suggest that the psychological effects of tDCS may depend not only on target selection but also on intervention dose and the specific cognitive-behavioral domain being addressed.

The findings for emerging tES modalities further illustrate why neurophysiological effects do not necessarily translate into immediate clinical benefit. This review included two studies examining tACS and HD-tIPNS (16, 20). Ahn et al. (16) found that tACS increased sensorimotor alpha power and that this increase was associated with reduced pain severity, suggesting modulation of pain-relevant brain rhythms. Adhia et al. (20) reported that HD-tIPNS altered connectivity within pain-related brain networks, but without concomitant behavioral improvement. These apparently discordant findings may reflect the fact that neurophysiological markers are often more sensitive to acute stimulation effects than clinical outcomes. In chronic pain, immediate modulation of oscillatory activity or functional connectivity may not be sufficient to overcome long-standing behavioral, affective, and sensorimotor dysfunction. Repeated stimulation, optimized dosing, or pairing with active rehabilitation may be required for neurophysiological changes to accumulate and eventually translate into clinically meaningful analgesia. Thus, the discrepancy between neural and clinical outcomes does not necessarily indicate inefficacy, but rather highlights a translational gap between short-term biological effects and measurable symptom improvement. As these observations are each limited to a single small-scale pilot study, they should be interpreted with caution and do not provide sufficient evidence to establish the clinical efficacy of tACS or HD-tIPNS for CNLBP. The mechanistic explanations offered above—regarding the translational gap between acute neurophysiological changes and clinical outcomes—are intended to contextualize these findings within broader neuromodulation theory rather than to claim direct support from the included trials.

An important methodological consideration across the included trials concerns the credibility of sham stimulation and the integrity of participant blinding. Active tDCS and tACS typically produce transient cutaneous sensations, including tingling, itching, or warmth under the electrodes, particularly during current ramp-up and ramp-down. Although all included studies reported sham stimulation and double-blind designs, sham procedures varied across the included trials: the majority employed current ramp-up and ramp-down to replicate initial cutaneous sensations while omitting sustained stimulation, whereas a minority used alternative approaches such as reduced current intensity or active sham configurations. This variability in sham design may introduce differential degrees of unblinding across studies. Only a minority formally assessed blinding integrity using standardized questionnaires or controlled for sensory differences between conditions. Participants may still discriminate between active and sham conditions based on the duration or quality of perceived sensations, and this potential imperfect blinding may influence subjective outcomes such as pain intensity, which are susceptible to placebo and nocebo effects. This possibility should be considered when interpreting the pooled estimates for pain reduction.

Risk of bias was assessed using the original Cochrane RoB 1 tool. This instrument was selected because the review protocol was established at an early stage using RevMan 5.4.1, which integrates RoB 1 as its standard module; changing to RoB 2 after protocol finalization would have introduced assessment inconsistency. Although RoB 2 is now the preferred approach for randomized trials, the core bias domains evaluated by RoB 1, random sequence generation, allocation concealment, blinding of participants and personnel, blinding of outcome assessors, incomplete outcome data, and selective reporting, correspond substantially to those assessed by RoB 2, so no critical domain was omitted. Furthermore, the two reviewers underwent joint calibration training using RoB 1 criteria; switching instruments after this calibration phase would have compromised inter-rater reliability. To examine whether studies at high risk of bias influenced the pooled estimates, we conducted a sensitivity analysis excluding trials with high risk of bias in two or more key domains. For the primary outcome of pain intensity in combined tDCS trials, the pooled effect estimate remained directionally consistent, suggesting that the findings are not disproportionately driven by high-risk studies. Future updates of this review will adopt RoB 2.

This study has several limitations. First, the number and quality of the included studies were variable; some studies had small sample sizes and were at risk of bias. Furthermore, as noted above, blinding of participants may be imperfect in some trials due to the inherent sensory differences between active and sham tES, and this potential limitation may influence subjective outcomes such as pain intensity. Second, substantial heterogeneity in intervention protocols, control conditions, and outcome measures limited the feasibility and interpretability of quantitative pooling. Third, follow-up durations were generally short; most trials assessed outcomes immediately post-treatment or within a one-month window, and very few included extended follow-up (e.g., 3 months or longer). This precludes robust conclusions regarding the durability of tDCS effects and whether the observed pain reductions represent transient neuromodulatory after-effects or sustained clinical improvement in a chronic condition. Fourth, psychological outcomes were assessed using diverse instruments and were often incompletely reported, which hindered consistent evaluation of effects across studies. Fifth, adverse event reporting was not standardized across included trials, and few studies employed systematic tolerability questionnaires or quantified dropout rates attributable to intervention-related adverse events, limiting the precision of the safety synthesis. Future research should address these limitations by conducting large, multicenter randomized controlled trials with standardized stimulation protocols and outcome measures; systematically comparing different stimulation targets and co-intervention pairings; exploring individualized stimulation strategies based on neuroimaging or electrophysiological characteristics; extending follow-up to at least 6 months; incorporating a broader range of functional and psychosocial outcomes; and using neuroimaging methods to investigate how tES interacts with pain-related neural networks during active rehabilitation or behavioral treatment.

## 5 Conclusion

In summary, current evidence is insufficient to support the clinical efficacy of tDCS as a standalone treatment for CNLBP. Evidence for other tES modalities (HD-tDCS and tACS) is limited to single exploratory studies and remains preliminary. When combined with behavioral or physical interventions, tDCS was associated with a statistically significant reduction in pain intensity; nevertheless, firm clinical conclusions are difficult to draw at this stage given the substantial heterogeneity across stimulation protocols, co-interventions, and outcome measures, as well as the limited sample sizes and variable methodological quality of the included trials. Improvements in disability and psychological outcomes remain limited and inconsistent. The differential findings across intervention groups may be explained by differences in stimulation target, treatment dose, co-intervention type, and the degree to which the paired intervention engages the neural systems modulated by stimulation. As the vast majority of available evidence derives from conventional tDCS protocols, with only single exploratory trials assessing HD-tIPNS and tACS, conclusions regarding tES as a broad modality class should be treated as preliminary. Further high-quality research is needed to clarify the optimal role of tDCS and other tES modalities in the comprehensive management of CNLBP.

## Data Availability

The original contributions presented in the study are included in the article/Supplementary material, further inquiries can be directed to the corresponding author/s.
