# Supplementary material for: Efficacy of transcranial electrical stimulation in the treatment of chronic nonspecific low back pain: a systematic review and meta-analysis
Source: Front Neurol. 2026 Aug 19;17:1797108. doi: 10.3389/fneur.2026.1797108 (PMC13533783; doi:10.3389/fneur.2026.1797108)
Supplement: Supplementary file 2 [file Table_1.DOCX]

Supplementary Material

# 1 Supplementary Data

Appendix 1 Database Search Strategy

| PubMed | ((((("Low Back Pain"[Mesh]) OR (chronic low back pain)) OR (chronic nonspecific low back pain)) OR (LBP)) OR (NSLBP)) AND ((((((("Transcranial Direct Current Stimulation"[Mesh]) OR (tACS)) OR (tRNS)) OR (tPCS)) OR (HD-tDCS)) OR (CES)) OR (Transcranial Electrical Stimulation)) |
| --- | --- |
| Web of Science | TS=((("low back pain" OR "chronic low back pain" OR "chronic nonspecific low back pain" OR LBP OR nsabp) AND ("transcranial direct current stimulation" OR tDCS OR tACS OR tRNS OR tPCS OR "HD-tDCS" OR CES OR "transcranial electrical stimulation"))) |
| ScienceDirect | TITLE-ABSTR-KEY(("low back pain" OR "chronic low back pain" OR LBP OR NSLBP OR "chronic nonspecific low back pain") AND ("transcranial direct current stimulation" OR tDCS OR tACS OR tRNS)) |
| Chinese Quarterly Virtual Information Platform (CQVIP) | (((((((题名或关键词=慢性腰痛 OR 题名或关键词=chronic low back pain) OR 题名或关键词=chronic lumbago) OR 题名或关键词=chronic lumbar pain) OR 题名或关键词=慢性下背痛) OR 题名或关键词=慢性腰背痛) OR 题名或关键词=慢性下腰痛) AND ((((题名或关键词=tDCS OR 题名或关键词=tACS) OR 题名或关键词=tRNS) OR 题名或关键词=经颅直流电刺激) OR 题名或关键词=电刺激)) |
| China National Knowledge Infrastructure（CNKI） | (主题:经颅电刺激 +经颅电刺激疗法)OR(篇关摘:经颅直流电刺激 +经颅直流电刺激治疗 + tACS + tRNS(精确))AND(主题:慢性腰痛 + 慢性腰痛患者 +'慢性腰痛(clbp)'+ 非特异性慢性腰痛) |
| WANFANG DATA | (（主题:(慢性腰痛) or 题名或关键词:(腰痛 or 非特异性腰痛)） and （主题:(经颅电刺激) or 题名或关键词:(经颅直流电刺激 or 经颅脉冲电流刺激 or 随机噪声刺激 or 经颅交流电刺激)) |

Appendix 2 Basic characteristics of the literature

| Author, Year | Experimental design type | Intervention measures | Intervention Number | Age | Participants | Other intervention measures | Primary results | Safety | Follow-up Schedule | Sham Procedure |
| --- | --- | --- | --- | --- | --- | --- | --- | --- | --- | --- |
| Adhia et al.,2022 | Parallel-group | HD-tIPNS | 1 | 41.9 | 30 | \ | NPRS | Common mild itching/tingling, no serious adverse events | Immediately post-intervention, 1 day post-intervention | 60s ramp-up and ramp-down |
| Ahn et al.,2019 | Crossover | tACS | 1 | 18–65 | 20 | \ | DVPRS | Not reported | Immediately post-intervention | 10s ramp-up and ramp-down |
| Corti& Loftus.,2022 | Parallel-group | tDCS | 8 | 53.16 | 19 | \ | VAS、RMDQ、PCS | Not reported | Immediately post-intervention | 30s ramp-up and ramp-down |
| Jiang et al.,2019 | Parallel-group | tDCS | 1 | 41.96 | 51 | \ | NRS | Common mild itching/tingling, no serious adverse events; only one reported severe dizziness but did not withdraw | Immediately post-intervention | 10s ramp-up and ramp-down |
| Mariano et al.,2019 | Parallel-group | tDCS | 10 | 63.1 | 21 | \ | DVPRS、WHY-MPI-C、RMDQ、PASS-10、CPAQ-8 | Generally good | Assessments on day 1, day 5, day 10 post-intervention; follow-up at 6 weeks after the last intervention | 30s ramp-up and ramp-down |
| Mcphee et al.,2021 | Crossover | HD-tDCS | 3 | 28.6 | 12 | \ | VAS、MPQ、RMDQ | Common mild itching/tingling, no serious adverse events; one subject terminated prematurely due to intolerable scalp pain | Immediately post-intervention, 4 days post-intervention, 2 weeks post-intervention | 60s ramp-up and ramp-down |
| O'Connell et al.,2013 | Crossover | tDCS | 9 | 45 | 8 | \ | VAS、RMDQ | Common mild itching/tingling, no serious adverse events | Baseline, daily during intervention, follow-up for 3 days post-intervention; final follow-up at 3 weeks after intervention end | 5s ramp-up and ramp-down |
| Alcon et al.,2025 | Parallel-group | tDCS | 5 | 41.4 | 20 | PNE | NPRS、MODI、PCS、TSK | Common mild itching/tingling (25%), no serious adverse events | Immediately post-intervention | 30s ramp-up and ramp-down |
| Armbrust et al.,2024 | Parallel-group | tDCS | 10 | 36.88 | 48 | OMT | VAS、RMDQ | Common mild itching/tingling, no serious adverse events (main adverse reactions during total course: mild tingling sensation 3.6%, mild headache 2.3%, mild drowsiness 1.9%; no skin burns) | Immediately post-intervention, 1 month post-intervention | 30s ramp-up and ramp-down |
| Diniz et al.,2026 | Parallel-group | tDCS | 10 | Over 18 | 60 | TENS | NRS、BPI、RMDQ | Not reported | 1 month post-intervention | 30s ramp-up and ramp-down |
| Ehsani et al.,2023 | Parallel-group | tDCS | 8 | 33 | 45 | CBT | RMDQ、TSK、PASS | Common mild itching/tingling, no serious adverse events | 1 month post-intervention | 10s ramp-up and ramp-down |
| Hazime et al.,2017 | Parallel-group | tDCS | 12 | 51.9 | 46 | PES | NRS、SF-MPQ、RMDQ | Common mild itching/tingling, no serious adverse events | 1 month, 3 months, 6 months post-intervention | No specific ramp-up and ramp-down process was set; stimulation was turned on for 30 seconds and then turned off |
| Jafarzadeh et al.,2019 | Parallel-group | tDCS | 6 | 31.58 | 36 | Postural training | VAS | Common mild itching/tingling, no serious adverse events | 1 month post-intervention | 10s ramp-up and ramp-down |
| Bo et al.，2022 | Parallel-group | tDCS | 5 | 40.23 | 74 | Exercise therapy | VAS、RMDQ | Common mild itching/tingling, no serious adverse events | Immediately post-intervention, post-exercise intervention, 1 month post-intervention | No specific ramp-up and ramp-down process was set; stimulation was turned on for 30 seconds and then turned off |
| Luedtke et al.,2015 | Parallel-group | tDCS | 5 | 44.5 | 122 | CBT | VAS、RAND-36、ODI | Common mild itching/tingling, no serious adverse events | Immediately post-intervention, post-CBT, 4 weeks, 12 weeks, 24 weeks | 8s ramp-up and ramp-down |
| Lu et al.，2024 | Parallel-group | tDCS | 12 | 36.4 | 40 | Exercise therapy | NRS、ODI、PCS | Not reported | Assessed only at pre-treatment and 4 weeks post-treatment; no long-term follow-up | 30s ramp-up and ramp-down |
| Sacca et al.,2025 | Parallel-group | HD-tDCS | 6 | 41 | 74 | Acupuncture | NRS | Not reported | Immediately post-intervention | 15s ramp-up and ramp-down |
| Schabrun et al.,2014 | Crossover | tDCS | 1 | 30 | 14 | PES | NRS | Not reported | Immediately post-intervention, 1 day post-intervention, 3 days post-intervention | 10s ramp-up and ramp-down |
| Straudi et al.,2018 | Parallel-group | tDCS | 5 | 55.1 | 35 | Exercise therapy | VAS、RMDQ | Common mild itching/tingling (82%), no serious adverse events | Post-tDCS, post-combined intervention, 1 month post-intervention | No specific ramp-up and ramp-down process was set; stimulation was turned on for 30 seconds and then turned off |

Note: Parallel-group RCT: Each participant receives only one type of stimulation; Crossover RCT: Each participant receives both active and sham stimulation or more than one type of stimulation, with a washout period of at least 7 days between adjacent stimulation sessions.

BPI: Brief Pain Inventory；CBT: Cognitive Behavioral Therapy；CPAQ-8: Chronic Pain Acceptance Questionnaire-8；DVRPS: Digital and Verbal Rating Pain Scale；HD-tDCS: High-Definition Transcranial Direct Current Stimulation；HD-TENS: High-Definition Transcutaneous Electrical Nerve Stimulation；MODI: Modified Oswestry Disability Index；MPQ: McGill Pain Questionnaire；NPRS: Numeric Pain Rating Scale；NRS: Numeric Rating Scale；ODI: Oswestry Disability Index；PASS-10: Pain Anxiety Symptoms Scale-10；PCS: Pain Catastrophizing Scale；PES: Percutaneous Electrical Stimulation；PNE: Pain Neuroscience Education；QMT: Quasi-Manual Therapy；RCT: Randomized Controlled Trial；RMDQ: Roland-Morris Disability Questionnaire；SF-MPQ: Short-Form McGill Pain Questionnaire；tACS: Transcranial Alternating Current Stimulation；tDCS: Transcranial Direct Current Stimulation；TENS: Transcutaneous Electrical Nerve Stimulation；TSK: Tampa Scale of Kinesiophobia；VAS: Visual Analog Scale；VRS: Verbal Rating Scale；WHY-MPI-C: Wisconsin Brief Pain Inventory-Chinese V

Appendix 3 The specific tables and their classifications

| **Behavior** | Biodex Balance System（including OSI， APSI， MLSI）：Berg Balance Scale (BBS)；Schober Test；Surface Electromyography (sEMG)；Root-Mean-Square Difference (RMSD)；Relative Area (RA)；Relative Width (RW)；Relative Height (RH) |
| --- | --- |
| **Cognitive function** | Stroop Color-Word Test (SCWT) / Stroop Color-Word Task；Comprehensive Trail Making Test 2 (CTMT2)；CTMT2 Inhibition Index；CTMT2 Set Shifting Index；Two-Choice Reaction Time Task (2CRT)；Four-Choice Reaction Time Task (4CRT)；Word Recognition Task (for Episodic Memory) |
| **Brain function** | Cerebral Blood Flow Indicators；EEG Functional Connectivity；Analysis Method: standardized low-resolution brain electromagnetic tomography (sLORETA)；Regional Activity Analysis；Instantaneous Phase Synchronization；Lagged Phase Synchronization；Effective Connectivity Analysis；EEG Alpha Oscillation Strength；Two-Point Discrimination Threshold |
| **Research process and safety** | Adverse Event Reporting；Simulation Results |
| **Patient-reported outcome** | SF-36 Health Survey (SF-36)；Patient Perceived Satisfactory Improvement (PPSI)；EuroQoL 5-Dimension 3-Level (EQ-5D-3L)；RAND-36 Health Survey (RAND-36)；Global Perceived Effect (GPE)；EuroQoL 5-Dimension (EQ-5D)；Credibility/Expectancy Questionnaire (CEQ)；Client Satisfaction Questionnaire-8 (CSQ-8)；Perception of Stimulation Type (Blinding Assessment) |

Appendix 4 List of Abbreviations

| Abbreviation | Full Term |
| --- | --- |
| BBS | Berg Balance Scale |
| BPI | Brief Pain Inventory |
| CBT | Cognitive Behavioral Therapy |
| CI | Confidence Interval |
| CLBP | Chronic Low Back Pain |
| CNLBP | Chronic Nonspecific Low Back Pain |
| CPM | Conditioned Pain Modulation |
| CPAQ-8 | Chronic Pain Acceptance Questionnaire-8 |
| CTMT2 | Comprehensive Trail Making Test 2 |
| dACC | Dorsal Anterior Cingulate Cortex |
| DLPFC | Dorsolateral Prefrontal Cortex |
| DVPRS | Defense and Veterans Pain Rating Scale |
| EEG | Electroencephalography |
| EMG | Electromyography |
| EQ-5D | EuroQol 5-Dimension |
| FABQ | Fear-Avoidance Beliefs Questionnaire |
| GAD-7 | Generalized Anxiety Disorder-7 |
| GPE | Global Perceived Effect |
| HADS | Hospital Anxiety and Depression Scale |
| HD-tDCS | High-Definition Transcranial Direct Current Stimulation |
| HD-tIPNS | High-Definition Transcutaneous Peripheral Nerve Stimulation |
| M1 | Primary Motor Cortex |
| MeSH | Medical Subject Headings |
| MODI | Modified Oswestry Disability Index |
| mPFC | Medial Prefrontal Cortex |
| MPQ | McGill Pain Questionnaire |
| NRS | Numeric Rating Scale |
| NPRS | Numerical Pain Rating Scale |
| ODI | Oswestry Disability Index |
| PASS | Pain Anxiety Symptoms Scale |
| PCS | Pain Catastrophizing Scale |
| PES | Peripheral Electrical Stimulation |
| pgACC | Pregenual Anterior Cingulate Cortex |
| PHQ-9 | Patient Health Questionnaire-9 |
| PNE | Pain Neuroscience Education |
| PRISMA | Preferred Reporting Items for Systematic Reviews and Meta-Analyses |
| PRO | Patient-Reported Outcome |
| PPT | Pressure Pain Threshold |
| RCT | Randomized Controlled Trial |
| RMDQ | Roland-Morris Disability Questionnaire |
| rTMS | Repetitive Transcranial Magnetic Stimulation |
| SAS | Self-Rating Anxiety Scale |
| SCWT | Stroop Color-Word Test |
| SDS | Self-Rating Depression Scale |
| sEMG | Surface Electromyography |
| SF-36 | 36-Item Short Form Health Survey |
| SF-MPQ | Short-Form McGill Pain Questionnaire |
| sLORETA | Standardized Low-Resolution Brain Electromagnetic Tomography |
| SMD | Standardized Mean Difference |
| tACS | Transcranial Alternating Current Stimulation |
| tDCS | Transcranial Direct Current Stimulation |
| tES | Transcranial Electrical Stimulation |
| TSK | Tampa Scale of Kinesiophobia |
| TSP | Temporal Summation of Pain |
| VAS | Visual Analogue Scale |
| VRS | Verbal Rating Scale |
| WHY-MPI-C | West Haven-Yale Multidimensional Pain Inventory |
| WMD | Weighted Mean Difference |

Appendix 5 Summary of Findings: GRADE Evidence Profile

| **Outcome** | **Comparison** | **Studies (participants)** | **Effect estimate (SMD, 95% CI)** | **I²** | **Limitations** | **Certainty** |
| --- | --- | --- | --- | --- | --- | --- |
| Pain intensity | Standalone tDCS vs sham | 5 (91) | −0.22 [−0.52, 0.08] | 0% | Imprecision (−1)¹ | Moderate |
| Pain intensity | Combined tDCS (M1) vs control | 11 (371) | −0.57 [−0.93, −0.21] | 81% | Inconsistency (−1)²; Indirectness (−1)³ | Low |
| Pain intensity | Combined tDCS (DLPFC) vs control | 2 (60) | −0.67 [−1.38, 0.04] | 79% | Very serious imprecision (−2)⁴ | Very low |
| Functional disability | Standalone tDCS vs sham | 4 (43) | −0.23 [−0.69, 0.22] | 0% | Imprecision (−1)¹ | Moderate |
| Functional disability | Combined tDCS (M1) vs control | 7 (202) | −0.14 [−0.63, 0.36] | 82% | Inconsistency (−1)²; Indirectness (−1)³ | Low |
| Functional disability | Combined tDCS (DLPFC) vs control | 3 (44) | −0.38 [−0.84, 0.07] | 0% | Imprecision (−1)¹ | Moderate |
| Pain-related psychological outcomes | Narrative synthesis (tDCS ± co-interventions) | 7 (~300) | Not pooled | NA | Inconsistency (−1)²; Indirectness (−1)³ | Low |
| Pressure pain threshold | tDCS vs sham/control | 3 (~80) | Mixed findings (not pooled) | NA | Inconsistency (−1)²; Imprecision (−1)¹ | Low |
| Pain intensity (tACS) | tACS vs sham | 1 (20) | Single exploratory pilot; reduced pain with increased alpha power | NA | Very serious imprecision (−2)⁴; Indirectness (−1)³ | Very low |
| Neurophysiological outcomes (HD-tIPNS) | HD-tIPNS vs sham | 1 (30) | Single exploratory pilot; altered connectivity without clinical pain reduction | NA | Very serious imprecision (−2)⁴; Indirectness (−1)³ | Very low |

**Footnotes**

1. **Imprecision:** Small sample size, wide confidence interval crossing or approaching the null, optimal information size not met.
2. **Inconsistency:** Substantial heterogeneity across studies (I² > 80% or heterogeneous outcomes/constructs precluding meta-analysis).
3. **Indirectness:** Combined interventions or outcome measures vary in clinical relevance and applicability; or single-study/surrogate outcomes limit generalizability.
4. **Very serious imprecision:** Very small number of studies (≤2) or single exploratory trial with very small sample size, critically insufficient information.

**Abbreviations:** CBT, cognitive behavioral therapy; CI, confidence interval; CNLBP, chronic nonspecific low back pain; DLPFC, dorsolateral prefrontal cortex; GRADE, Grading of Recommendations Assessment, Development and Evaluation; HD-tIPNS, high-definition transcranial infraslow pink-noise stimulation; M1, primary motor cortex; OMT, osteopathic manipulative treatment; PNE, pain neuroscience education; SMD, standardized mean difference; TENS, transcutaneous electrical nerve stimulation; tACS, transcranial alternating current stimulation; tDCS, transcranial direct current stimulation; tES, transcranial electrical stimulation.
